# COVID-19 and household water insecurities in vulnerable communities in the Mekong Region

**DOI:** 10.1007/s10668-022-02182-0

**Published:** 2022-02-25

**Authors:** Louis Lebel, Hap Navy, Phoummixay Siharath, Chau Thi Minh Long, Nilar Aung, Phimphakan Lebel, Chu Thai Hoanh, Boripat Lebel

**Affiliations:** 1 Unit for Social and Environmental Research, Department of Social Science and Development, Faculty of Social Sciences, Chiang Mai University, Chiang Mai, Thailand; 2 Inland Fisheries Research and Development Institute, Fisheries Administration, Ministry of Agriculture, Forestry and Fisheries, Phnom Penh, Cambodia; 3 Department of Environmental Engineering, Faculty of Engineering, National University of Laos, Vientiane, Lao PDR; 4 Western Highlands Agriculture and Forestry Science Institute, Dak Lak, Vietnam; 5 University of Yangon, Yangon, Myanmar; 6 International Water Management Institute, Regional Office for Southeast Asia, Vientiane, Lao PDR

**Keywords:** Water insecurity, COVID-19, Vulnerable groups, Mekong region, SDG 6

## Abstract

Access to sufficient clean water is important for reducing the risks from COVID-19. It is unclear, however, what influence COVID-19 has had on water insecurities. The objective of this study was to assess the associations between COVID-19 control measures and household water insecurities. A survey of 1559 individuals living in vulnerable communities in five countries (Cambodia, Laos, Myanmar, Thailand, Vietnam) showed that increased needs for clean water to wash hands or facemasks made it more likely a person was water insecure along those dimensions. Water insecurities with respect to handwashing and drinking, in turn, made adoption of the corresponding good practices less likely, whereas in the case of washing facemasks there was no association. Water system infrastructure, environmental conditions such as floods and droughts, as well as gender norms and knowledge, were also important for water insecurities and the adoption of good practices. As domestic water insecurities and COVID-19 control measures are associated with each other, efforts should therefore be directed at identifying and assisting the water insecure at high risk when COVID-19 reaches their communities.

**Supplementary Information:**

The online version contains supplementary material available at 10.1007/s10668-022-02182-0.

## Introduction

Access to sufficient clean water for drinking, preparing food, hygiene, and sanitation is important for reducing risks of COVID-19 transmission in vulnerable populations and for health outcomes if a person is infected (Amankwaa & Fischer, 2020; Desye, 2021; Lau et al., 2020; Stoler et al., 2020). Household water insecurities arise when the ability to obtain and benefit from water for household uses is reduced or threatened, for example, by problems of availability, access, or allocation (Gupta & Lebel, 2020; Jepson et al., 2017; Young et al., 2021). Poor water quality is another source of insecurity, for example, as a major cause of diarrhoea (Hannah et al., 2020). Common measures to prevent the spread of COVID-19 may influence household water insecurities in several ways. First, frequent handwashing (Lao et al., 2021; Maude et al., 2021), and wearing reusable cloth facemasks (Bauza et al., 2021; Duong et al., 2021; MacIntyre et al., 2021), may lead to growing demand for water in homes where clean water access is already restricted at certain times (Sayeed et al., 2021; Sempewo et al., 2021; Zvobgo & Do, 2020). Second, lockdowns and road closures may disrupt the delivery of drinking water or disrupt the servicing of water systems (Desye, 2021; Howard, 2021; Neal, 2020). Third, the closure of businesses and markets reduces incomes, pushing people towards cheaper but less safe water sources (Shao et al., 2021). Finally, an increased need for water in situations where pump outlets or delivery points are shared make conventional social distancing guidelines impractical, increasing the risks of COVID-19 transmission (Hasan et al., 2021; Stoler et al., 2021).

The combined impacts of water insecurities and COVID-19 measures may be particularly severe for residents of urban informal settlements without their own water supplies (Corburn et al., 2020; Wilkinson, 2020), communities in remote locations with limited infrastructure (Eichelberger et al., 2021), refugee camps with inadequate facilities (Rafa et al., 2020), or migrant worker dormitories and camps with shared facilities and no spatial independence (Djalante et al., 2020; Kim et al., 2021). Such communities are often already vulnerable because of power relations and discrimination (Stoler et al., 2020; Wilkinson, 2020). In addition, within communities, women and girls may be more vulnerable to the impacts, as they often carry the greater burden for water collection, cleaning, and care-giving (Adams et al., 2021). The elderly, bed-ridden, or disabled may also be especially vulnerable, as they are dependent on others for access (Scherer et al., 2021).

Recognizing that pre-existing water insecurities may make the impacts of COVID-19 outbreaks more severe, and vice versa, COVID-19 impacts may make water insecurities more serious. In this study, we assess the drivers and consequences of household water insecurities in vulnerable communities in five countries in the Mekong Region (Cambodia, Laos, Myanmar, Thailand, Vietnam), in the context of an unfolding COVID-19 pandemic and on-going efforts to meet Sustainable Development Goal 6 (SDG 6)—‘*ensure availability and sustainable management of water and sanitation for all’* (UNESCAP, 2021). In mid-2020, a ranking of 47 countries in the Asia–Pacific Region according to a COVID-19 water security risk rating (Guthrie & Roiko, 2020), ranked three of the Mekong countries in the top 10 at highest risk: Laos (3rd), Cambodia (6th), and Myanmar (7th). Vietnam ranked 26th and Thailand 36th. The countries cover a range of water, sanitation, and hygiene (WASH) development contexts. Laos, for instance, had the fewest COVID-19 infections and deaths (Phonvisay et al., 2021), but also the lowest coverage of handwashing facilities in rural areas (Table SM1). Thailand, in contrast, had the highest rate of COVID-19 infections, but the highest coverage of handwashing facilities. Clearly, the relationship between COVID-19 and water insecurities are more nuanced than what aggregate national rankings or statistics can reveal.

Studies in Vietnam in more privileged communities suggest that COVID-19 led to more frequent handwashing and switch to hand sanitizers when water and soap were not available (Huong et al., 2020), and daily washing of cloth facemasks when subjects did not opt for single-use disposable surgical masks (Duong et al., 2021). Water insecurity has not, however, been examined closely in otherwise relevant studies of COVID-19 impacts in more vulnerable and potentially water insecure communities in the Mekong Region. Thus, a study in ethnic minority upland communities in Chiang Rai, Thailand, documented positive changes in hygiene practices such as wearing facemasks in public spaces and frequent handwashing, but also problems from reduced income and limited access to the health care system (Kitchanapaibul et al., 2021). In a companion paper, coping responses were also documented (Suratana et al., 2021). Neither analysis mentioned water.

**Fig. 1:**
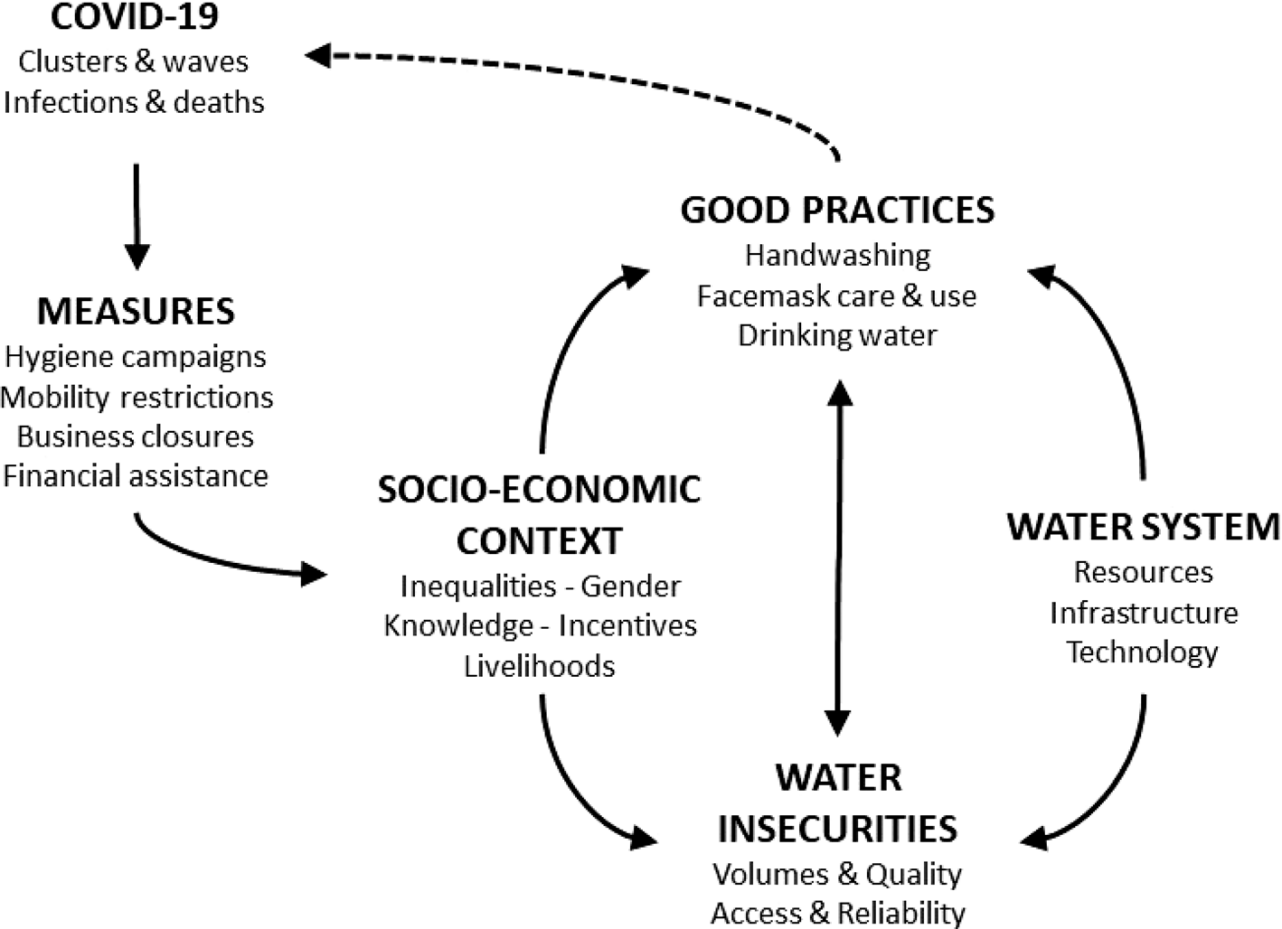
Key relationships of interest in this study

From previous studies in the Mekong Region, it is therefore unclear what influence common control measures have had on household water insecurities, and whether water insecurities influence the adoption of good practices (Fig. 1). In this study, we chose to focus on water used for washing hands, drinking, and washing facemasks, as these three uses span different water sources and practice routines, and thus likely different patterns of association with COVID-19. We address two questions.

1. How did the COVID-19 outbreak effect household water insecurities in vulnerable communities?
2. How did household water insecurities impact the adoption of practices important to reducing the risks of COVID-19 infection?

## Methods

### Site and informant selection

In each country, communities in which all or most homes were not connected to a central public water supply system (local community schemes were included) were selected from administrative areas in three geographic zones: upland rural, lowland rural or peri-urban, and urban informal settlements (Table SM2). Several representative communities likely to be water insecure were chosen within each area after consultation with public health offices or local governments. Households and informants within communities were selected by convenience sampling. Most interviews were collected between 20 January and 21 April 2021, while a few in Myanmar were delayed because of travel restrictions and not completed until 2 June 2021.

### Survey instrument

The survey instrument covered basic information about the respondent, their sources of drinking water and handwashing water, access problems, household washing facilities, handwashing and facemask washing practices, impacts of COVID-19 outbreak, gender division of labour and decisions, social well-being, as well as needs for further assistance (See Supplementary Materials). As the survey was carried out among marginalized groups, we kept the format of most questions as simple as possible—most commonly requiring either a Yes or No answer. The survey instrument was translated into five national languages, with an occasional further step of translation in the field to a minority language where necessary. Pre-tests were done in all countries, and the project team met online several times to ensure the intended meaning of each question was understood and captured in the survey instrument in all languages. Respondents gave their verbal consent to participate and were provided with compensation or gift to cover their time.

The survey instrument was developed using the online software Alchemer (formerly SurveyGizmo). Answers from respondents were normally entered directly onto hand-held tablets or smartphones by a trained interviewer, except where there was no reliable access to internet at a field site, in which case results were recorded first on paper and later uploaded.

### Key measures

Unlike work aimed at developing a coherent, standardized set of questions for comparing and tracking changes in household water insecurities (Tsai et al., 2015; Young et al., 2019), this study focused on designing measures for a one-off survey around three specific, contrasting household water uses of importance to COVID-19 transmission in vulnerable communities using multiple water sources of varying quality. The key measures used in this study to characterize a respondent in relation to handwashing, drinking, or facemask washing are defined in Table SM3. Being *handwashing water insecure* (HWWI), for example, was measured by combining responses to 8 questions covering four aspects: whether water quality was perceived as adequate (2), water volumes sufficient to needs (1), water supply improving (4), and water-related intra-household conflicts (1) (Table SM3). For aspects with multiple question, items responses were weighted so total was 1 for each aspect (or -1 in case of reliability as a reversed scale). We opted for simple questions (Yes or No), based on respondent’s experience (rather than summarizing across all members of a household) whenever possible, and a long recall period of over a year, so we could cover with a single survey both the wet and dry seasons known to be very important for water quality and volumes in our target study areas. The long recall period also allowed for some differences in the timing of COVID-19 impacts in different countries. One limitation of the HWWI measure is that it does not include information on severity, for instance, duration or frequency of periods where did not have enough clean water or water system performed poorly. The HWISE scale using a recall period of four weeks (Young et al., 2019) allows for a more detailed questions and nuanced measure of insecurities, but in this setting would have required multiple surveys in different seasons and national pandemic ‘waves’ that were beyond resources available for this study. The items used in this study included items referring to water quality, an aspect not explicitly addressed in the 12-item HWISE scale (Young et al., 2019).

Three measures of good water practices, one corresponding to each insecurity item, were also defined (Table SM3). *Good handwashing practices* (GHWP), for example, combined identifying situations where washing was important with responses to questions about number of times per day, with time spent each time, and changes in practices in response to COVID-19 (Table SM3). The main limitation of this measure is that it did not include information about the washing process or steps.

Derivations of other composite measures used in the analysis, such as financial insecurity, health insecurity, and social insecurity, are given in Table SM3.

### Data analysis

Analyses of the associations between binary outcome variables and predictors of special interest adjusted for potential confounding variables were done using logistic regression models (Hosmer & Lemeshow, 2000). The strengths of association were measured and interpreted using odds ratios—abbreviated to OR in this paper if adjusted, and to OR_raw_ if not adjusted. Odds ratios greater than one indicate an increased likelihood, whereas ratios smaller than one indicate a decreased likelihood of the outcome. Where appropriate a 95% confidence interval (CI_95_) for the odds ratio estimate is provided. A CI_95_ that does not include 1 can be interpreted as being statistically significant from the baseline group.

Two types of statistical models were built following the logic of our conceptual framework (Fig. 1) and sequence of research questions. The first, assessed the impacts of COVID-19 on the likelihood of being water insecure, while considering the social context and water system. The second, assessed the association of water insecurities with the adoption of good practices that could help reduce risks of COVID-19 transmission.

The modelling approach was to initially force the inclusion of predictors of special interest, and to identify other candidate predictors using automated backward elimination option in SPSS25 software, and in a second step to manually simplify the model further, retaining only significant (*P* < 0.05) predictors. All candidate predictor variables used were categorical with transformation of scores and indices with many values to ordinal variables with 3–5 levels to reduce influence of outliers, and so that nonlinear associations could be easily detected without needing to conduct a series of tests, and to keep interpretation of odds ratios simple and consistent in form across predictors. In interpreting significant associations, further analysis was done to better understand associations among closely related variables and individual indicators in composite measures.

## Results

The findings are organized according to the framework (Fig. 1), starting with information about the vulnerable communities studied and how their lives were impacted by COVID-19. The water sources for drinking and washing are then briefly summarized before the analysis moves onto the two main sections of the results, focussed on the sources of water insecurities and the adoption of practices that reduce the risks of COVID-19 transmission.

### Vulnerable communities

Basic characteristics of respondents and their households are given in Table 1. A few noteworthy points include that only 13% of respondents had a regular salary, while 42% worked as daily labourer, and 40% had difficulties in making loan repayments, underlining the financial precariousness of many in these communities. Although respondents were living in remote locations, peri-urban transition zones, or in urban informal settlements, 85% had been in residence at their current location for more than 10 years (Table 1), underlining that these vulnerable communities are not transitory.

**Table 1:** Basic characteristics of respondents and their households (n = 1559)

Characteristic	Categories	%		Characteristic	Categories	%
Gender	Women Men	56.8 43.2		Migrant	Yes No	8.3 91.7
Age (years)	< 19 20–29 30–39 40–49 50–59 60 +	3.0 14.5 26.6 23.8 19.3 12.8		Residence time (years)	< 1 1–5 6–10 11–20 20 +	2.1 6.7 6.4 16.7 68.1
Education	No formal Some primary Some secondary Higher	18.1 35.7 22.0 24.2		Loan repayment	Difficulties No difficulties No loan	40.3 21.4 38.3
Key assets^a^	Car / Pickup	18.5		Income sources^a^	Daily wage labour	41.6
	Motorcycle	85.4			Selling goods	21.6
	Television	78.9			Farming/fishing	39.8
	Fridge	47.1			Regular salary	12.8
	Mobile phone	91.7				

^a^ not mutually exclusive, household members

A total of 53 respondents (3.4%) surveyed claimed to have ‘never heard of COVID-19’ and thus were not asked questions that assume at least some minimal awareness. A comparison with those who had heard of COVID-19 indicates the former are much more likely to have had no formal education (OR_raw_ = 9.12; CI_95_:5.21–16.2), belonged to an ethnic minority group (OR_raw_ = 14.9; CI_95_:6.68–33.2), and lived in upland as opposed to urban sites (OR_raw_ = 16.3 CI_95_:4.88–68.4). Unaware respondents were found at sites in Laos (7.7%), Thailand (7.9%), and Myanmar (1.0%). This group of COVID-19 unaware individuals is a vulnerable group that unfortunately could not be included in the main statistical models reported.

#### COVID-19 disrupts lives

**Fig. 2:**
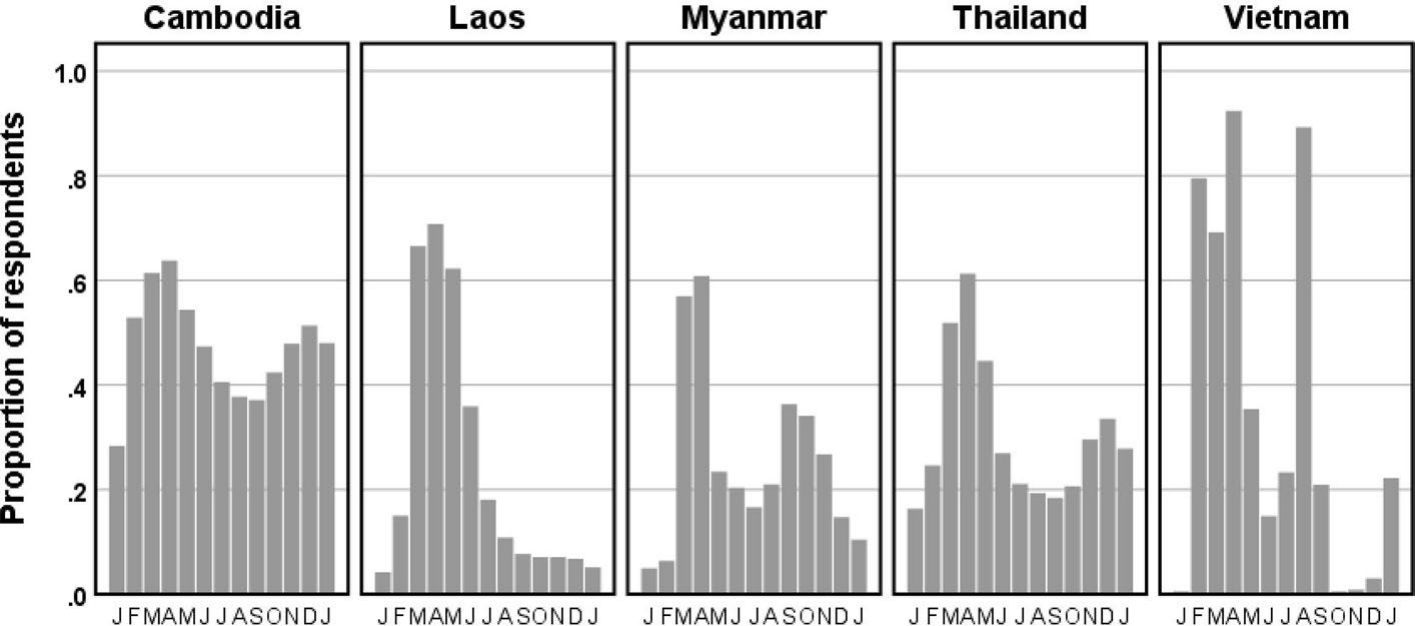
Months in which livelihoods, mobility, and access to goods and services were significantly disrupted by COVID-19 outbreak and control measures. Mean proportion of respondents in each country for the period January 2020 through January 2021

At the time of interviews (February–May 2021), only 8% of households had a member who had been tested or undergone quarantine for COVID-19. Nevertheless, COVID-19 measures such as mobility restrictions and closing of certain businesses had significant impacts on livelihoods, mobility, and access to goods and services over most of 2020 (Fig. 2). The patterns across countries were similar in the first six months of 2020, corresponding to the first wave, but diverged more in the following six months or start of the second wave (Fig. 2). The specific activities impacted followed similar patterns in the five countries with a few exceptions. Respondents in Laos, for instance, were less likely to have experienced reduced income, whereas those in Vietnam suffered more from restrictions on mobility, and those in Myanmar most from loss of access to goods and services (Figure SM1).

#### Water sources for drinking and washing purposes

**Fig. 3:**
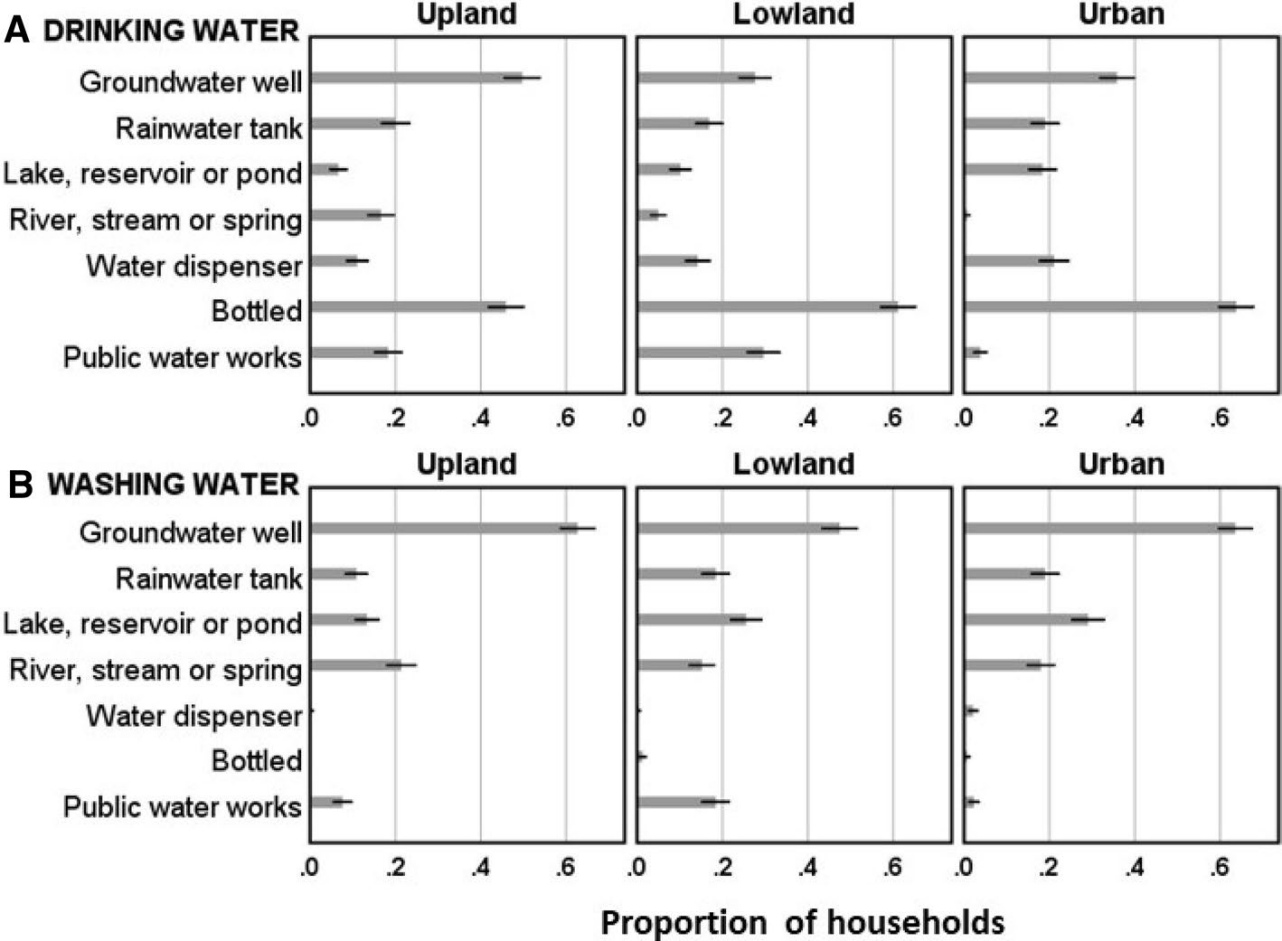
Sources of drinking (**a**) and washing (**b**) water of households in three ecological zones. Means and 95% confidence intervals

Across the five countries, the most common source of water for hand and facemask washing was groundwater wells (58%). For drinking water, bottled sources ranked highest overall (56%). In the Mekong Region, ‘bottled’ includes not just small (< 1 L) bottles but also larger reusable plastic containers of 10–20 L. There were some important differences by ecological zone, with groundwater wells being more important in upland sites for drinking, and bottled water relatively less important (Fig. 3a). For sources of drinking water other than bottled or from a dispenser, most respondents boiled and/or filtered water prior to consumption. Thus, of the 8% who drank from running surface waters, 84% boiled or treated water beforehand, while of the 33% who drank well water, a similar proportion (82%) boiled or treated. In lowland and upland sites, water for hand and facemask washing sometimes came from local or central waterworks systems (Fig. 3b), but in many of these cases (77%) washing water was still carried back to the home, implying water was not piped directly to individual homes. Households in the urban informal settlement zones were rarely connected to the public waterworks system (2%). Many households had multiple sources of water for handwashing (32%) and drinking (46%).

### Sources of household water insecurities

Being *handwashing water insecure* (HWWI, 54%), *drinking water insecure* (DWI, 49%), or *facemask washing water insecure* (FWWI, 33%) was defined by combining responses to questions about problems experienced in the past year with the quantity and quality of water for each use, as well as trends in reliability of supply (Table SM3). Just under half (48%) of respondents, for example, had experienced not having enough clean water to drink in the past year. Differences among zones in the problems faced were modest, with residents in lowland sites, for example, encountering dirty or unsafe water more often than in other zones (Figure SM3). In the rest of this section, logistic regression models (Table 2) are used to mutually adjust association of various predictors with the three forms of household water insecurity.

**Table 2:** Associations between the impacts of COVID-19 on practices and household water insecurities, adjusting for different water conditions and social contexts. Entries in body of table are odds ratios (and 95% confidence intervals) from three logistic regression models. Baseline for comparison, if not shown explicitly with ‘1’, is the absence or opposite of the stated condition

Predictor variables	Handwashing Water Insecure (HWWI)	Drinking Water Insecure (DWI)	Facemask Washing Water Insecure (FWWI)
*COVID-19 impacts on practices*			
Washed hands more thoroughly	1.79 (1.12–2.85)	–	–
Switched to cleaner sources	0.56 (0.43–0.73)	–	–
Drank more from unsafe sources	–	3.71 (2.51–5.48)	–
Less money spent on drinking water		1.46 (1.12–1.90)	
Wore mask more outside the home	–	–	2.06 (1.24–3.42)
Washed masks more thoroughly	–	–	2.15 (1.42–3.25)
Washed masks more frequently	–	–	2.33 (1.58–3.44)
*Water system and conditions*			
Multiple hand washing stations	–	0.65 (0.48–0.88)	–
Must carry washing water to home	1.63 (1.26–2.11)	1.46 (1.10–1.94)	–
Surface water source	2.28 (1.65–3.16)	2.89 (1.93–4.32)	–
Rainwater tank source	–	2.81 (1.85–4.26)	–
Groundwater well source	–	–	2.26 (1.51–3.37)
Conflict over water resources	–	1.44 (1.02–2.03)	–
Salinization of water	2.25 (1.34–3.78)	–	–
Drought has impacts	1.85 (1.41–2.43)	–	–
*Socio-economic context*			
Gender role in water supply: Men Shared Women	1 1.32 (0.99–1.74) 2.06 (1.42–2.99)	1 1.65 (1.24–2.19) 1.32 (0.91–1.90)	1 2.49 (1.82–3.40) 3.35 (2.28–4.93)
Gender role in food and care: Men Shared Women	1 3.40 (1.91–6.08) 2.33 (1.35–4.02)	–	–
Socially insecure: Low Mid High	1 1.51 (1.12–2.03) 1.54 (1.16–2.04)	–	1 1.44 (1.04–1.99) 2.02 (1.50–2.73)
Health insecure: Low Mid High	1 1.68 (1.27–2.22) 1.33 (0.98–1.80)	–	–
Financially insecure: Low Mid High	–	1 0.85 (0.65–1.11) 0.57 (0.38–0.85)	–
Multiple information sources	0.70 (0.54–0.92)	–	–
Zone			
Upland Lowland Urban	–	–	1 2.16 (1.59–2.93) 0.66 (0.49–0.91)
Country: Cambodia Laos Myanmar Thailand Vietnam	1 0.50 (0.31–0.81) 1.10 (0.69–1.76) 0.57 (0.33–1.00) 2.63 (1.59–4.37)	1 5.44 (3.59–8.24) 3.23 (1.97–5.27) 1.80 (1.19–2.74) 1.54 (1.00–2.39)	1 0.33 (0.20–0.54) 2.95 (1.81–4.81) 1.08 (0.66–1.76) 1.13 (0.73–1.75)
Nagelkerke	*R*^2^ = 0.25	*R*^2^ = 0.31	*R*^2^ = 0.31

#### COVID-19 impacts

There were a few associations between changes in practices, which respondents attributed to the impacts of the COVID-19 outbreak and being water insecure (Table 2). Respondents who had increased the thoroughness of handwashing (94%) because of the COVID-19 outbreak were twice as likely to be HWWI (OR = 1.79), and those who washed facemasks more frequently (OR = 2.33) or more thoroughly (OR = 2.15) were likewise more likely to be FWWI (Table 2). In all cases, the increased attention to hygiene implies an increased need for clean water; finding significant associations with insecurity measures implies that the availability of sufficient clean water is not guaranteed.

Changes among water sources of different quality were also important to water insecurity. Thus, switches to cleaner sources for handwashing (36%) in response to COVID-19 outbreak made it less likely (OR = 0.56) of being HWWI, while drinking water more frequently from unsafe sources (12%) made it more than three times (OR = 3.71) more likely of being DWI (Table 2). These associations are consistent with the importance of water quality indicators in the definition of all three water insecurities (Table SM3).

Increased wearing of facemasks outside the home (90%) was also associated with FWWI, consistent with the increased frequency and intensity of facemask washing. Respondents with less money to spend on drinking water (39%) were more likely to be DWI (OR = 1.46).

#### Water systems

In terms of water micro-infrastructure, if water must be carried to the home, then a respondent was more likely to be handwashing (OR = 1.63) or drinking (OR = 1.46) water insecure (Table 2). Having access to more handwashing stations reduced the likelihood of being drinking water insecure (OR = 0.65). Convenient access to clean water is important to reducing water insecurities.

Respondents were more than twice as likely (OR = 2.28) to be HWWI if household sources included surface waters (rivers or ponds). Almost a third (31%) had at some time used surface sources to wash their hands (Fig. 3b). If supplies were affected by droughts (49%) or salinization (6%), respondents were more likely to be HWWI (Table 2). Dry season droughts and salinization reduce the volume and quality, respectively, of water for washing hands and contribute to HWWI. Respondents were more likely to be DWI if household drinking water sources included surface waters (OR = 2.89) or rainwater tanks (OR = 2.81). Using groundwater wells for washing also made it more likely for a respondent to be FWWI (OR = 2.26). Unimproved or ‘natural’ sources are of unreliable quality and their use thus a potential source of water insecurity.

#### Gender norms

**Fig. 4:**
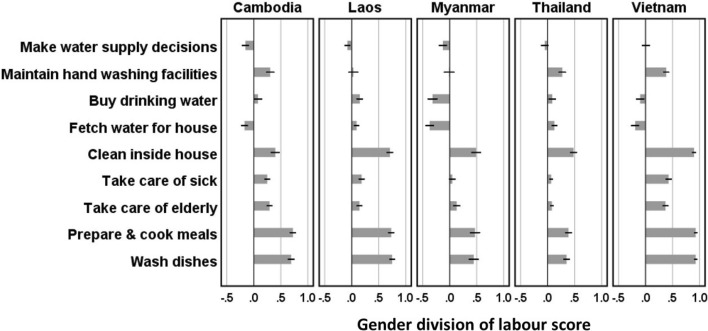
Gender division of labour in the household. Scores vary from women dominate role (+ 1) through both or neither (0) to men dominate role (− 1)

Households in which women had a similar (shared) or a relatively greater role than men in managing water supplies (Fig. 4) were more likely to be handwashing and facemask washing water insecure (Table 2). One possible explanation is that members of such households are more aware of needs for clean water because women are primary users in the home (Fig. 4), and therefore more likely to detect water insecurities. Looking more closely at specific responsibilities, one of the strongest individual associations was between households in which women (as opposed to men) were responsible for maintaining handwashing facilities and being HWWI (OR_raw_ = 3.11; CI_95_:2.28–4.24). Overall, women are burdened much more than men by roles in food preparation, dish washing, cleaning inside the house, and care-giving tasks (Fig. 4). Many of these activities involve washing hands. Thus, households in which women dominate food and care roles were also more likely to be HWWI (Table 2).

An alternative explanation to awareness or perception of insecurities is that households in which women dominate water supply management are less able to address water insecurities, as this requires influence or power within and beyond the household, and this is often held by men. Survey evidence does not support the within household argument. Households in which women (as opposed to men) make water supply decisions were more likely to be HWWI (OR_raw_ = 1.77; CI_95_:1.26–2.51) and FWWI (OR_raw_ = 2.56; CI_95_:1.77–3.71). We did not collect information that could be used to test the beyond the household argument.

#### Socio-economic context

There were a few other significant associations with socio-economic factors. Respondents with many information sources were less likely than those with few sources to be HWWI (Table 2). This suggests experience or knowledge is important to reducing insecurities. Without adjustment for other predictors, those with a high education were less likely to be HWWI (OR_raw_ = 0.77; CI_95_:0.61–0.97), whereas those with more than 20 years at current location were more likely to be water insecure (OR_raw_ = 1.31; CI_95_:1.12–1.53) than those with fewer than 5 years residence, implying it is an issue of knowledge or information rather than experience. Obtaining information about COVID-19 from social media channels was common (Figure SM2) and was associated with being less likely to be HWWI (OR_raw_ = 0.69; CI_95_:0.56–0.85), whereas those who obtained information from health workers were more likely to be HWWI (OR_raw_ = 1.61; CI_95_:1.31–1.98), perhaps because they set stricter standards.

Respondents that were more socially insecure were more likely to be FWWI and HWWI (Table 2), implying that social relations are important for access to water for washing. Increased tension within the household due to COVID-19, for example, made it more likely to have experienced not having enough clean water to wash facemasks (OR_raw_ = 2.59; CI_95_:2.01–3.33). Respondents that were more financially insecure were less likely to be DWI (Table 2), counter to expectations that drinking water access would be more of a problem for those with reduced income or less wealth. Respondents with intermediate health insecurity scores were significantly more likely to be HWWI than those with low scores.

Respondents in urban areas were less, and those in lowlands more, likely than those in uplands to be FWWI. Seasonal drought impact household water uses in the uplands (46%) more often than in urban areas (28%). Patterns of water insecurities among countries were diverse (Table 2).

### Risk reduction practices

In this section, logistic regression models (Table 3) are used to mutually adjust association of various predictors with the adoption of *good handwashing practices* (GHWP, 26%), *good drinking water practices* (GDWP, 23%), and *good facemask washing and using practices* (GFWUP, 31%) as defined in Table SM3.

**Table 3:** Associations of water insecurities with good practices adjusted for water system and social context variables

Predictor variables	Good Handwashing Practices (GHWP)	Good Drinking Water Practices (GDWP)	Good Facemask Washing and Using Practices (GFWUP)
**Water Insecurity**			
Handwashing water insecure			
Low	1	–	–
Mid	0.69 (0.50–0.95)		
High	0.70 (0.49–0.98)		
Drinking water insecure			
Low	–	1	–
Mid		0.77 (0.55–1.09)	
High		0.56 (0.37–0.85)	
Facemask washing water insecure			
Low	–	–	1
Mid			1.03 (0.72–1.46)
High			1.16 (0.78–1.72)
**Water system and conditions**			
Multiple hand washing stations	1.67 (1.15–2.42)	–	–
Must carry washing water home	–	–	0.70 (0.53–0.93)
Multiple water sources	–	2.94 (2.05–4.24)	0.45 (0.29–0.68)
Rainwater tank source	0.16 (0.08–0.35)	–	–
Floods	1.47 (1.02–2.12)	2.94 (2.05–4.24)	–
*Socio-economic context*			
Woman respondent	1.58 (1.18–2.11)	–	–
Gender role in water supply			
Men Shared Women	1 1.11 (0.79–1.55) 1.86 (1.21–2.86)	1 0.62 (0.45–0.86) 0.35 (0.22–0.54)	–
Health insecurity score			
Low	1	–	–
Mid	1.33 (0.96–1.84)		
High	1.82 (1.26–2.62)		
Asset wealth higher	–	1.48 (1.07–2.05)	–
Higher education	–	1.79 (1.29–2.49)	1.36 (1.01–1.82)
Provided help to other households	1.49 (1.11–2.00)	–	1.57 (1.20–2.04)
Many information sources	–	–	1.95 (1.46–2.59)
Zone			
Upland Lowland Urban	1 1.45 (1.03–2.03) 1.60 (1.14–2.23)	1 0.60 (0.42–0.86) 1.40 (1.01–1.95)	–
Country			
Cambodia	1	1	1
Laos	7.32 (4.69–11.5)	0.38 (0.23–0.65)	2.60 (1.72–3.93)
Myanmar	1.04 (0.57–1.91)	0.05 (0.02–0.11)	3.17 (1.93–5.20)
Thailand	3.09 (2.05–4.67)	0.73 (0.47–1.14)	10.4 (6.81–15.9)
Vietnam	0.17 (0.08–0.36)	1.03 (0.64–1.66)	0.77 (0.44–1.35)
Nagelkerke	R^2^ = 0.37	R^2^ = 0.32	R^2^ = 0.32

Entries in body of table are odds ratios (and 95% confidence intervals) from logistic regression models. Baseline for comparison, if not shown explicitly with ‘1’, is the absence or opposite of the stated condition

#### Water insecurities

Respondents experiencing mid or high levels of *handwashing water insecure* (HWWI) were less likely to adopt GHWP than those with low insecurity scores (Table 3). Experiencing high levels of *drinking water insecure* (DWI) made it less likely (OR = 0.56) to adopt GDWP. Water insecurity in the case of handwashing and drinking was an obstacle to the adoption of good practices. In contrast, respondents experiencing intermediate or high levels of *facemask washing water insecure* (FWWI) were neither more nor less likely to have adopted GFWUP than those at a low level (Table 3).

#### Water systems

In terms of water systems, respondents were more likely (OR = 1.67) to follow GHWP if they had access to more handwashing stations (Table 3). Multiple facilities are an indicator that a house has water hygiene micro-infrastructure, making it easier to follow good practices. Having to carry washing water home, implying a household does not have a direct piped connection, made it less likely (OR = 0.70) to adopt GFWUP. These associations are consistent with the convenient access explanation noted earlier with respect to water insecurities.

Having had drinking water supplies impacted by floods was associated with a greater likelihood (OR = 2.94) of adopting GDWP (Table 3). Floods through contaminated run-off may reduce water quality. Residents with experience of being impacted by floods thus were much more likely to boil water prior to drinking (OR_raw_ = 6.42; CI_95_:4.98–8.29), and in doing so meet one of the conditions of being GDWP (Table SM3). Respondents having multiple water sources (OR = 2.94) for drinking were also more likely to adopt GDWP (Table 3).

Respondents impacted by floods were more likely to adopt GHWP (Table 3). Floods might help renew groundwater and surface water sources, reducing water shortages for washing hands. However, the evidence from our survey does not support this interpretation, whereby households impacted by floods were more likely to not have enough clean water to wash hands (OR_raw_ = 1.74; CI_95_:1.40–2.17). Those who had used rainwater from tanks for handwashing (16%) were less likely to adopt GHWP. One explanation for the latter association might be the amount that could be stored was quite limited and prioritized for drinking. Consistent with this interpretation is that of the 21% whom had used collected rainwater, 88% had done so for drinking. Moreover, those who used rainwater for washing hands were twice as likely to not have enough clean water to wash hands (OR_raw_ = 2.12; CI_95_:1.61–2.78).

#### Gender norms

Women were more likely (OR = 1.58) to adopt GHWP than men, while for GDWP and GFWUP there were no differences (Table 3). Households in which women had a greater role in securing water supplies were more likely to adopt GHWP, but less likely to follow GDWP than households in which men had the greater role (Table 3). One reason for the association with GHWP might be the better understanding of good practices regarding washing, cleaning, and food preparation that women have because social norms reinforce these work burdens on them (Fig. 4). That the association with GDWP is different may reflect that buying drinking water is often a shared (37%) or male (26%) responsibility.

#### Socio-economic context

People who were health insecure, after adjustment for other predictors, were more likely to follow GHWP (Table 3). The health insecure by definition (Table SM3) has recent experiences of being hospitalized (OR_raw_ = 5.00 CI_95_:3.91–6.36) or feeling unwell, which may inform or motivate good practices. Having multiple information sources or a higher education was associated with the adoption of GFWUP (Table 3). Television was the most important channel of information about COVID-19, but social media was also important, as were neighbours (Figure SM2). Households that provided help to other households to cope with the impacts of COVID-19 were more likely to have also adopted GHWP and GFWUP (Table 3). Patterns of association with zones were complex. Residents in urban and lowland sites were more likely to adopt GHWP than those in upland sites, perhaps reflecting less exposure to public health campaigns in remote locations. People in urban sites were more likely to obtain information from government officials than those in upland sites (OR_raw_ = 1.42; CI_95_:1.08–1.88), providing partial support for this explanation. Those in lowland sites, however, were less likely, and in urban sites more likely, to adopt GDWP than those in upland sites.

## Discussion

Government measures to control the spread of COVID-19 involved restricting movement or limiting business activities, disrupted livelihoods and reduced incomes in vulnerable groups, but had few traceable impacts on household water insecurities. Behavioural campaigns aimed at hygiene practices, like washing hands and wearing and washing facemasks, had stronger associations with water insecurities, as they increased needs for clean water in a context in which it may be seasonally in short supply, low in quality, or difficult to access in vulnerable communities. Studies in other low- and middle-income countries in other parts of the world have also found that risk-reducing practices require access to more clean water, and this may not be easily available (Sempewo et al., 2021; Stoler et al., 2021; Zvobgo & Do, 2020).

Household water insecurities had mixed associations with the adoption of good practices for reducing risks of COVID-19. Being water insecure with respect to handwashing, made the adoption of good handwashing practices less likely—as noted above, perhaps reflecting difficulties in meeting increased demand for clean water. Being water insecure with respect to drinking water also made the adoption of good treatment practices less likely. Being drinking water insecure was strongly associated with the relatively rare use of unsafe surface water sources. The COVID-19 outbreak resulted in increased consumption from these unsafe sources, as well as more boiling prior to drinking. In contrast to the first two insecurities, being facemask washing water insecure was not significantly associated with good facemask washing and using practices. This may reflect strong cultural, as well as mandatory requirements to wear facemasks in public, regardless of whether daily washing or replacement of facemasks is feasible.

More broadly, differences between washing hands and masks are reflected in routines. Washing hands happens several times a day, including instances that are not scheduled, whereas washing masks happens less frequently and is scheduled, and may be combined with washing clothes. This suggests convenient access may be more important for handwashing than facemask washing—something that depends on micro-infrastructure or facilities such as buckets, taps, hoses, and soap holders. Drinking water happens many times a day, but preparation (boiling and other treatments) may be done just once a day, while buying bottled water might be done weekly. Moreover, handwashing is an individual activity, whereas washing facemasks or securing clean drinking water might be done by one person for other household members. This study was one of the first studies to report on the significance of water access issues for washing facemasks—with COVID-19 having impacts on water insecurity.

Water systems are important to insecurities and practices. A lack of household water infrastructure, as indicated by need to carry handwashing water home, was associated with being water insecure, while having multiple places to wash hands was strongly associated with good hand and facemask washing practices. We suggest that convenience is a key factor in compliance with public health guidance. Our findings are consistent with other studies and reviews which find that adequate facilities and infrastructure to support water, sanitation, and hygiene practices are important to reducing risks from major health crises such as the COVID-19 pandemic (Bauza et al., 2021; Desye, 2021).

Environmental conditions, in particular floods and droughts, had implications for the performance of water systems, sometimes contributing to water insecurities and the adoption of good practices. Water quality issues were often key, underlining the importance of continuing to actively pursue SDG 6 targets in the Mekong Region countries by focussing efforts on vulnerable, water insecure communities. In the context of the COVID-19 pandemic, greater attention is needed on disposal of solid wastes and wastewater (Islam et al., 2021; Tortajada, 2021).

Social context was important for water insecurities and hygiene practices, not just infrastructure and technologies. Women were more likely than men to adopt good handwashing practices. A study in Vietnam also found women had better handwashing practices than men (Huong et al., 2020). A study in Indonesia also documented that women were more likely to comply with voluntary COVID-19 measures, in part because it fit with norms on gender roles (Paramita et al., 2021). Two norms were examined in this study. The first was in responsibility for securing water supplies for the household—overall this was evenly balanced between men and women and often shared. The second was in responsibilities for food preparation, dishwashing, and care-giving—this was strongly skewed towards women and often their sole responsibility. Households in which women had a greater role (burden) with respect to the managing of water supplies were more likely to be water insecure with respect to washing hands, drinking water, and washing facemasks. There is some evidence consistent with the idea that women are more aware of water insecurities because they are primary users of water within the household. Other possible explanations include women in such households may be too busy to fetch more water for washing, or that men are less likely to assist when water use is for an activity they don’t normally do (or feel any responsibility for), and thus not seen as a priority.

In the vulnerable communities surveyed in this study, beliefs in the protective merits of frequent handwashing and wearing facemasks were high and comparable to other studies of the general population (Huong et al., 2020), healthcare workers (Maude et al., 2021), or students (Duong et al., 2021) in the Mekong Region. While most people in the Mekong Region had access to multiple sources of information, there were some significant knowledge gaps in some remote ethnic minority and migrant communities studied, with some individuals effectively unaware of the COVID-19 outbreak at the time of interviews. This points to communication failures. Information about COVID-19 prevention in this study mostly came from television, while government officials were only a common source in Cambodia. Social media was also a significant source in all countries. More work is needed to understand the role of different stakeholders—governmental and non-state—have played in providing information and responding with actions to address problems of water insecurity and COVID-19 transmission risks in vulnerable communities.

Differences among countries, after adjustment for other variables, were often substantial. Respondents from the Vietnamese sites in the Central Highlands, for example, were more likely to be water insecure with respect to handwashing water than respondents from most other countries, and were less likely to adopt good handwashing practices. In this case, the challenges from seasonal drought that impacts even groundwater wells are an important shared factor across sites. Good drinking water practices were least likely in Myanmar, reflecting a still high reliance on unimproved water sources. Across countries, dependence on wells for drinking water, for instance, was greater in the uplands than in urban or lowland areas where bottled water predominated.

This study had some important limitations. First, as a cross-sectional survey we had to rely on respondents recall and attributions to assess impacts. In addition, the recall period was long, which meant we could only ask simple questions about experiences and perceptions. Second, the key measures of water insecurity and good practices were not comprehensive. We did not look closely at handwashing techniques or facemask handling and disposal practices, nor did we cover all dimensions of household water uses. Third, this study did not examine sanitation issues. Given the importance of water quality problems in these vulnerable communities, this was a significant gap. Future work should also look more closely at the associations between drinking water sources and sanitation.

## Conclusions

In conclusion, the findings of this study show that household water insecurities and COVID-19 measures may influence each other. Changes in practices made in response to the COVID-19 outbreak exacerbated water insecurities, while water insecurities were an obstacle to adoption of some good practices. In the Mekong Region, water quality issues were often a prominent aspect of insecurities. Water systems, from sources through to household micro-infrastructure, are diverse and important to insecurities and the adoption of good practices. Gender norms assign different roles to women and men in relation to procuring versus using water in the household.

Based on our findings, we offer a couple of suggestions for policy development and program design. First, greater attention should be given by governments and non-governmental organizations to identify water insecure households in vulnerable communities, as they are likely to be at high risk when COVID-19 reaches their communities. Communities which, for various reasons, are often not adequately served by programs aimed at the general population.

Second, programs promoting good hygiene practices for reducing COVID-19 risks should not assume that abundant clean water is always available, that all water used by a household comes from a single source, and that a source has a fixed quality. Programs need to be designed with attention to seasonality.

Third, in looking for ways to help vulnerable communities, consideration should be given to supporting micro-infrastructure that makes access to clean water more convenient. Convenient access reduces water insecurities and makes good hygiene practices important to COVID-19 responses more likely.

Fourth, while women often share with men responsibilities for securing household water supplies, women are the main users of water within the home for preparing food, washing dishes, cleaning, and care-giving. Aligned with these differences in roles, women are more likely than men to adopt good handwashing practices. COVID-19 risk reduction interventions targeting men may be needed to address these differences in perception and practice.

Taken together, these suggestions imply a combination of hardware and software is needed in vulnerable communities to effectively and simultaneously address household water insecurities and behaviours that reduce the risks of COVID-19 infection. Future research should examine interventions by state and non-state actors to reduce water insecurities, and how they have been impacted by COVID-19.

## Supplementary Information

Below is the link to the electronic supplementary material.

Supplementary file1 (DOCX 458 kb)
