# Supplementary material for: COVID-19 and household water insecurities in vulnerable communities in the Mekong Region
Source: Environ Dev Sustain. 2022 Feb 25;25(4):3503–22. doi: 10.1007/s10668-022-02182-0 (PMC8874302; doi:10.1007/s10668-022-02182-0)
Supplement: Supplementary file 1 — Supplementary file1 (DOCX 458 kb) [file 10668_2022_2182_MOESM1_ESM.docx]

**SUPPLEMENTARY MATERIALS**

**Table SM1** Selected national-level indicators

|  | Covid-19 incidence (per million population) | Basic drinking water services in rural areas (%) | Handwashing facilities in rural areas (%) | Basic sanitation services in rural areas (%) | Mortality rate due to unsafe water & sanitation (per 100,000) |
| --- | --- | --- | --- | --- | --- |
| Cambodia | 5,412 | 72.9 | 59.7 | 48.3 | 6.5 |
| Laos | 1,739 | 75.6 | 40.6 | 63.5 | 11.3 |
| Myanmar | 6,914 | 76.9 | 73.8 | 59.3 | 12.6 |
| Vietnam | 3,608 | 92.6 | 82.2 | 77.7 | 1.6 |
| Thailand | 15,071 | 100 | 83.2 | 98.3 | 3.5 |

^a^ Calculated based on number of infections reported on John Hopkins website on 23 August 2021 with population and all other information from World Bank Indicators.

**Table SM2** List of study sites. HWWI=handwashing water insecure; DWI=drinking water insecure; FWWI=facemask washing water insecure

| Site | Study Area | HWWI | DWI | FWWI | No Formal Education | Water works | Ethnic Minority | Drought | Floods | Pollution |
| --- | --- | --- | --- | --- | --- | --- | --- | --- | --- | --- |
| BD | Buôn Đôn District, Daklak Province, Vietnam | 76 | 39 | 66 | 25 | 67 | 5 | 94 | 68 | 24 |
| EK | Ea Kar District, Daklak Province, Vietnam | 78 | 30 | 37 | 16 | 2 | 19 | 97 | 84 | 17 |
| KB | Krông Bông District, Daklak Province, Vietnam | 88 | 63 | 56 | 13 | 0 | 18 | 100 | 100 | 23 |
| CM | Chiang Mai Municipality, Chiang Mai Province, Thailand | 50 | 27 | 17 | 10 | 13 | 17 | 35 | 32 | 16 |
| MW | Mae Wang District, Chiang Mai Province, Thailand | 46 | 56 | 30 | 25 | 55 | 95 | 94 | 3 | 45 |
| SS | Sansai District, Chiang Mai Province, Thailand | 37 | 41 | 41 | 75 | 29 | 97 | 13 | 16 | 4 |
| TG | Taunggyi District, Myanmar | 79 | 66 | 27 | 4 | 32 | 100 | 78 | 50 | 44 |
| PD | Pyapon District, Ayerarwady Region, Myanmar | 51 | 60 | 83 | 0 | 47 | 0 | 22 | 8 | 32 |
| SY | South Yangon District, Myanmar | 55 | 91 | 22 | 2 | 2 | 9 | 11 | 8 | 14 |
| SR | Siem Reap City, Siem Reap Province, Cambodia | 37 | 23 | 34 | 16 | 3 | 0 | 47 | 36 | 33 |
| BS | Banteay Srei District, Siem Reap Province, Cambodia | 31 | 13 | 25 | 28 | 1 | 0 | 46 | 34 | 26 |
| SN | Sotnikum District, Siem Reap Province, Cambodia | 55 | 35 | 24 | 16 | 4 | 0 | 57 | 36 | 33 |
| NV | Nava village, Toulikom District, Vientiane Province, Laos | 52 | 58 | 14 | 3 | 2 | 10 | 7 | 7 | 3 |
| PK | PhoukhaoKhouy, Toulikom District, Vientiane Province, Laos | 42 | 69 | 13 | 22 | 1 | 98 | 14 | 5 | 0 |
| PN | PhonNgam, Toulikom District, Vientiane Province, Laos | 39 | 69 | 10 | 9 | 0 | 1 | 12 | 7 | 0 |

**Table SM3** Definition of key measures of domestic water insecurity and good practices and other aggregated variables used in this study. See last section of Supplementary Materials for full questionnaire

| Measure | Derivation |
| --- | --- |
| Hand washing water insecure (HWWI) | *Since the beginning of last year until now,*  Q26 did you ever not have enough clean water to wash your hands when you wanted to do so (Yes=1)  Q27 did you ever wash your hands with water that you thought was dirty or unsafe (Yes=0.5)  Q28 did you ever stop washing hands with water from a source because it tasted, looked, or smelt bad (Yes=0.5)  Q29 was your supply of clean water for washing hands more reliable than in previous years (Yes=-0.25)  Q30 did a shortage of water for washing hands, ever create difficulties between you and other members of your household (Yes=1)  *Has the supply of water for washing hands changed such that the:*  Q35a volume of water normally available has increased (Yes=-0.25)  Q35b quality of water normally available has improved (Yes=-0.25)  Q35c reliability of water supply has improved (Yes=-0.25)  If total score >0 then HWWI |
| Drinking water insecure (DWI) | Since the beginning of last year until now,  Q21 did you ever not have enough clean water to drink when you wanted to do so (Yes=1)  Q22 did you ever have to drink water that smelt, looked, or tasted bad (Yes=1)  Q23 was your supply of clean drinking water more reliable than in previous years (Yes=-1)  Q24 did you ever feel uncomfortable or embarrassed about giving drinking water to visitors (Yes=1)  If total score >0 then DWI |
| Facemask washing water insecure (FWWI) | Since the beginning of last year until now,  Q42 did you ever not have enough clean water to wash your face mask when you wanted to do so (Yes=1)  Q43 did you ever wash your face mask with water that you thought was dirty or unsafe (Yes=0.5)  Q44 did you ever stop washing your face masks with water from a source because it tasted, looked or smelt bad (Yes=0.5)  If total score >0 then FWWI |
| Good hand washing practices (GHWP) | Q37 times per day washed hands (3 times or more)  Q38 time spent washing hands (10 seconds or more)  Q39 a list of 8 situations where wash hands (7 or more)  Did the COVID-19 outbreak have the following impacts (3 or more):  Q54a wash hands more thoroughly  Q54b wash hands more frequently  Q54c use soap more frequently  Q54d use alcoholic gel more frequently  If all four conditions were true then GHWP |
| Good drinking water practices (GDWP) | if drank water from following source (Q19) then treated it first (Q20) (6 or more)  public water works  bottled water  water from dispenser  river, stream or spring  lake, reservoir or pond  rain water tank  ground water well  Q56.4 boiled water more often after COVID-19  Q56.3 did not drink from less safer sources after COVID-19  If all three conditions were true then GDWP |
| Good facemask washing & use practices (GFWUP) | Q40 a list of 6 situations where wear mask (all 6)  Q41 reusable cloth facemask AND  Q60.4 washed masks more thoroughly after COVID-19  Q60.5 washed masks more frequently after COVID-19  Q41 disposable masks AND  Q60.6 changed masks more frequently after COVID-19  If all three conditions hold then GFWUP |
| Gender role in water supply | Q10.1 number males in household (>0) AND  Q10.2 number females in household (>0) AND  Sum of four tasks  Q65.1 fetch water for uses in home (women=1, men=-1, both or neither=0)  Q65.5 buy drinking water (women=1, men=-1, both or neither=0)  Q65.6 maintain handwashing facilities (women=1, men=-1, both or neither=0)  Q65.9 make decisions related to water supplies (women=1, men=-1, both or neither=0)  Divided by 4 |
| Gender role in food & care | Q10.1 number males in household (>0) AND  Q10.2 number females in household (>0) AND  Sum of four tasks  Q65.2 wash dishes (women=1, men=-1, both or neither=0)  Q65.3 prepare and cook meals (women=1, men=-1, both or neither=0)  Q65.7 take care of elderly members (women=1, men=-1, both or neither=0)  Q65.8 take care of sick or injured members (women=1, men=-1, both or neither=0)  Divided by 4 |
| Social insecurity score | Q63.2 Improved cooperation with others in the community (Yes=-1)  Q63.3 Made me more scared of strangers (Yes=1)  Q63.4 Increased tension or conflict within the household (Yes=1)  Q61.3 Neighbors or other households in your community helped (Yes=-1)  Q61.4 family or relatives in your community helped (Yes=-1)  Q61.5 Family or relatives living elsewhere helped (Yes=-1)  Q62 Helped other households in your community (Yes=-1)  Q18.2 ‘I have many friends’ applies to you (Yes=-1)  Q18.3 ‘I am well connected’ applies to you (Yes=-1)  Sum all the above |
| Health insecurity score | Q17 Hospitalize for illness or injury (Yes=1)  Q16 household member hospitalized for illness or injury (Yes=1)  Q15 member of household test or quarantined for COVID-19 (Yes=1)  Q18.1 ‘I often feel unwell’ applies to you (Yes=1)  Q18.4 ‘I often feel frustrated’ applies to you (Yes=1)  Q18.7 ‘I often feel trapped’ applies to you (Yes=1)  Q18.8 ‘I worry a lot’ applies to you (Yes=1)  Q18.6 ‘I feel fortunate’ applies to you (Yes=-1)  Q18.5 ‘I am hopeful about the future’ applies to you (Yes=-1)  Q48.5 COVID-19 made it more difficult to go to hospital or clinic (Yes=1)  Sum of above |
| Financial insecurity score | Q14 You or household member had difficulty repaying a loan (Yes=1)  Q12.1 You or household member own a car or pickup (Yes=-1)  Q13.6 You had a regular salary (Yes=-1)  The COVID-19 outbreak had following significant impacts on you  Q50.1 reduced my income (Yes=1)  Q50.2 made it harder to sell things (Yes=1)  Q50.3 increased cost of inputs (Yes=1)  Q50.4 reduced demand for services (Yes=1)  Q50.5 did fewer days work (Yes=1)  Sum of above |

**Fig. SM1** Impacts from COVID-19 outbreak control measures on mobility, access to goods and services, and livelihoods. Mean and 95% confidence intervals for proportion of respondents experiencing each impact


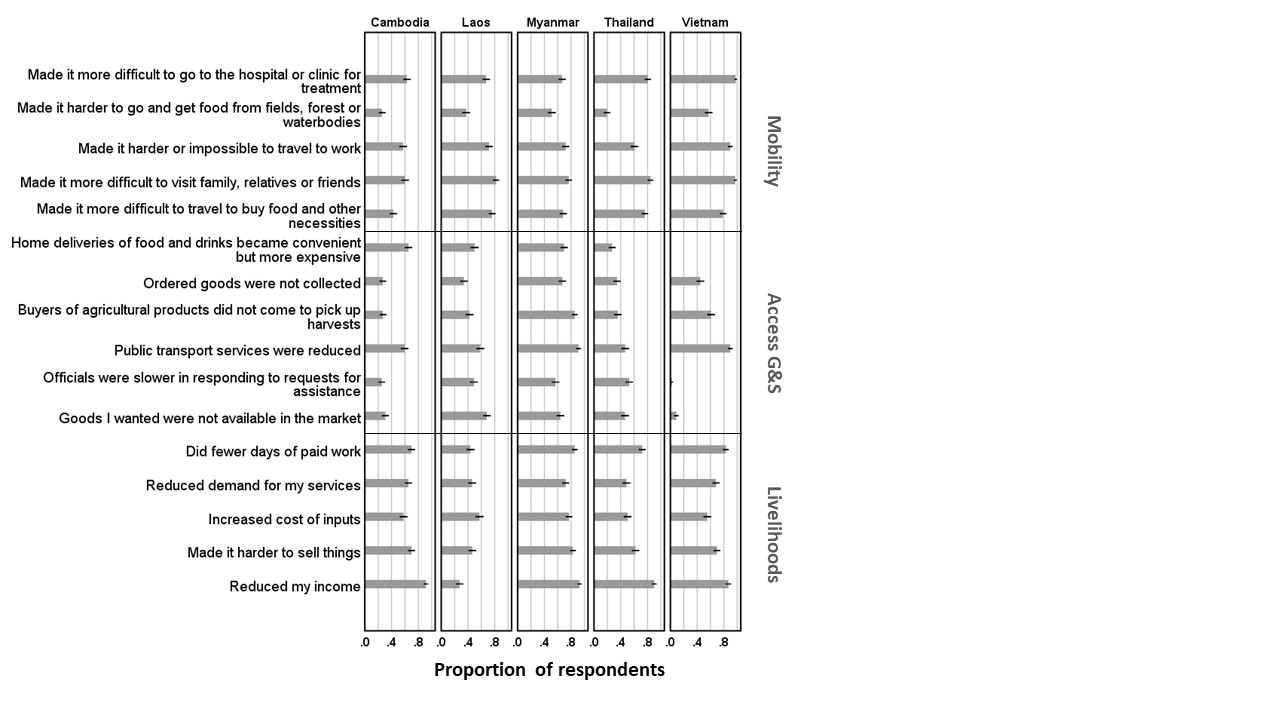


**Fig. SM2** Information sources and channels on COVID-19. Proportion of respondents


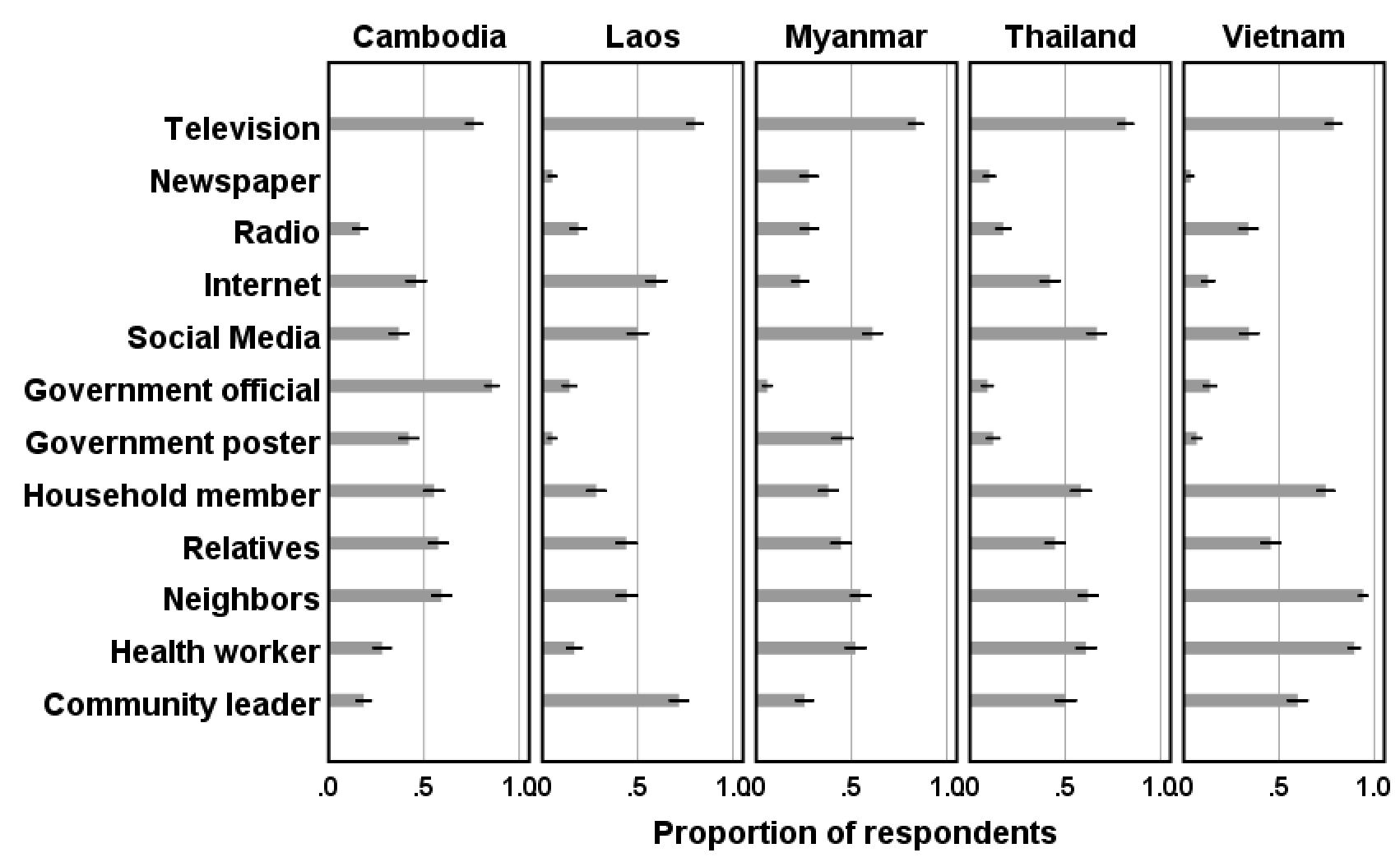


**Fig. SM3** Proportion of respondents experiencing specific problems with drinking, handwashing or facemask washing water access in three ecological zones


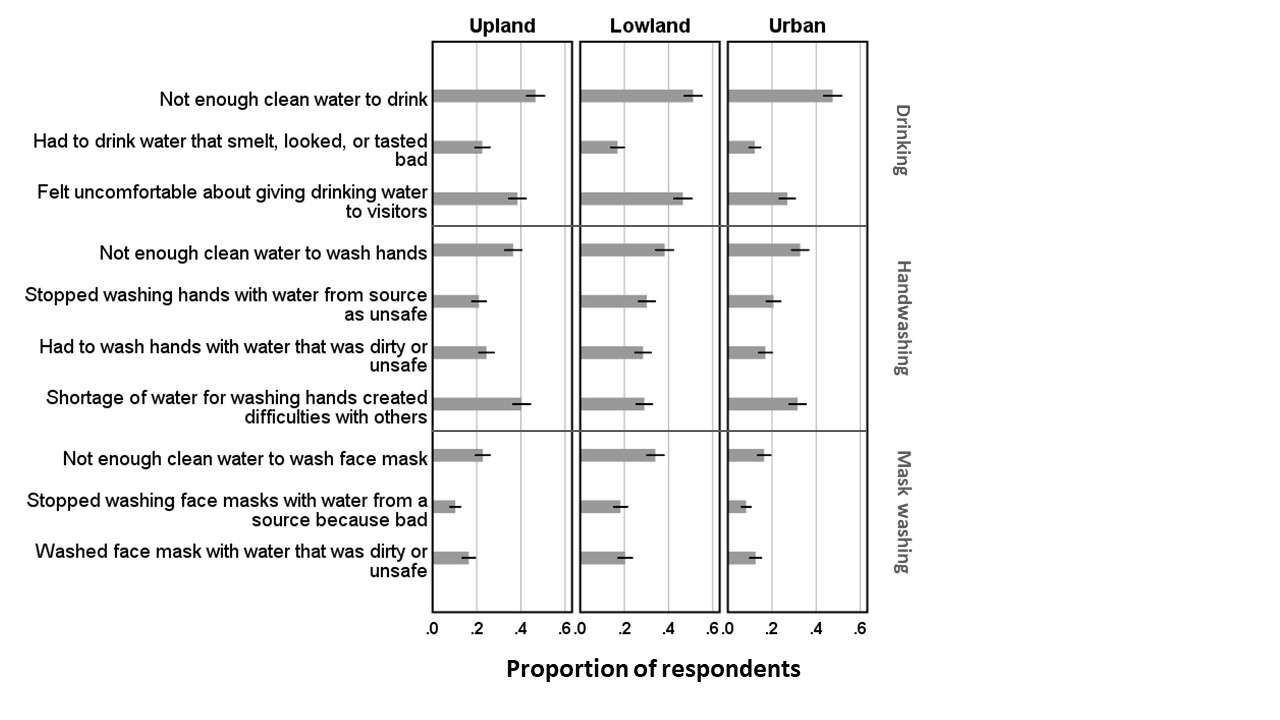


**Survey Instrument 1**

## About you

#### 1) Gender*

( ) Male

( ) Female

( ) Other

#### 2) How old are you?*

( ) <15

( ) 15-19

( ) 20-29

( ) 30-39

( ) 40-49

( ) 50-59

( ) 60+

#### 3) Of which countries are you a citizen? (You may select more than one box)*

[ ] Country 1

[ ] Country 2

[ ] Other - Write In: _________________________________________________

[ ] No formal citizenship

#### 4) What is your ethnicity? (You may select more than one box)*

[ ] Group 1

[ ] Group 2

[ ] Group 3

[ ] Other - Write In (Required): _________________________________________________*

#### 5) What religion do you follow?*

( ) Religion 1

( ) Religion 2

( ) Religion 3

( ) Other - Write In: _________________________________________________

#### 6) What is the highest level of education that you completed?*

( ) No formal education

( ) Incomplete Primary

( ) Primary School

( ) Vocational Education

( ) Lower Secondary School

( ) Upper Secondary School

( ) Undergraduate Degree

( ) Postgraduate Degree

#### 7) Since the beginning of last year until now, did you earn an income from any of the following sources? (You may select more than one box)*

[ ] Daily wage labor

[ ] Regular salary

[ ] Selling goods

[ ] Farming

[ ] Providing a service

[ ] Remittances from family

[ ] Other - Write In: _________________________________________________

## Where you live

### 8) Where is your household located?*

#### District*

( ) District 1

( ) District 2

( ) District 3

#### Country*

( ) Cambodia

( ) Laos

( ) Myanmar

( ) Thailand

( ) Vietnam

#### 9) How long ago did you first come to live at this location?*

( ) less than 3 months

( ) 3-12 months

( ) 1-5 years

( ) 6-10 years

( ) 11-20 years

( ) more than 20 years

## About your household

### 10) How many of the people, including yourself, that normally live in your household are the following?

Male: _________________________________________________

Female: _________________________________________________

Other: _________________________________________________

#### 11) Are you the head of this household?*

( ) Yes

( ) No

#### 12) Do you or other members of the household own any of the following? (You may select more than one box)*

[ ] Car or pickup

[ ] Motorcycle

[ ] Boat

[ ] Smart phone

[ ] Ordinary mobile phone

[ ] Fridge

[ ] Television

[ ] Other motorized vehicle

#### 13) Since the beginning of last year until now, did you or other members of your household earn income from the following sources? (You may select more than one box)*

[ ] Selling agricultural produce

[ ] Selling fish or other aquatic animals

[ ] Working on a farm

[ ] Daily wage laborer

[ ] Selling food or drink

[ ] Salary from private company

[ ] Salary from government

[ ] Other - please specify: _________________________________________________

#### 14) Since the beginning of last year until now, did any members of your household have difficulties repaying a loan?*

( ) Yes

( ) No

( ) Not applicable (no loan)

#### 15) Have any members of your household been tested or been quarantined for COVID-19?*

( ) Yes

( ) No

#### 16) Since the beginning of last year until now, apart from yourself, were any of the other members of your household hospitalized for any illness or injury?*

( ) Yes

( ) No

## Your wellbeing

#### 17) Since the beginning of last year until now, were you hospitalized for any illness or injury?*

( ) Yes

( ) No

#### 18) Do these statements apply to  you?*

|  | **Yes** | **No** |
| --- | --- | --- |
| I often feel unwell | ( ) | ( ) |
| I have many friends | ( ) | ( ) |
| I am well connected | ( ) | ( ) |
| I often feel frustrated | ( ) | ( ) |
| I am hopeful about the future | ( ) | ( ) |
| I feel fortunate | ( ) | ( ) |
| I often feel trapped | ( ) | ( ) |
| I worry a lot | ( ) | ( ) |

## Drinking water

#### 19) Since the beginning of last year until now, what were the sources of your drinking water? (You may select more than one box)*

[ ] Public water works

[ ] Bottled

[ ] Water dispenser

[ ] River, stream or spring

[ ] Lake, reservoir or pond

[ ] Rainwater tank

[ ] Groundwater well

[ ] Other - Write In: _________________________________________________

#### 20) Since the beginning of last year until now, did you treat (boil or filter) before drinking water from the following sources? (You may select more than one box)*

[ ] Public water works

[ ] Bottled

[ ] Water dispenser

[ ] River, stream or spring

[ ] Lake, reservoir or pond

[ ] Rainwater tank

[ ] Groundwater well

[ ] Other - Write In: _________________________________________________

[ ] Never boil or filter

#### 21) Since the beginning of last year until now, when at home, did you ever not have enough clean water to drink when you wanted to do so?*

( ) Yes

( ) No

#### 22) Since the beginning of last year until now, did you ever have to drink water that smelt, looked, or tasted bad?*

( ) Yes

( ) No

#### 23) Since the beginning of last year until now, was your supply of clean drinking water more reliable than in previous years?*

( ) Yes

( ) No

#### 24) Since the beginning of last year until now, did you ever feel uncomfortable or embarrassed about giving drinking water to visitors?*

( ) Yes

( ) No

## Handwashing water

#### 25) Since the beginning of last year until now, did you ever wash your hands with water from the following sources?*

[ ] Public water works

[ ] Bottled

[ ] Water dispenser

[ ] River, stream or spring

[ ] Lake, reservoir or pond

[ ] Rainwater tank

[ ] Groundwater well

[ ] Hand sanitizer

#### 26) Since the beginning of last year until now, when at home, did you ever not have enough clean water to wash your hands when you wanted to do so?*

( ) Yes

( ) No

#### 27) Since the beginning of last year until now, did you ever wash your hands with water that you thought was dirty or unsafe?*

( ) Yes

( ) No

#### 28) Since the beginning of last year until now, did you ever stop washing hands with water from a source because it tasted, looked or smelt bad?*

( ) Yes

( ) No

#### 29) Since the beginning of last year until now, was your supply of clean water for washing hands more reliable than in previous years?*

( ) Yes

( ) No

#### 30) Since the beginning of last year until now, did a shortage of water for washing hands, ever create difficulties between you and other members of your household?*

( ) Yes

( ) No

## Handwashing facilities

#### 31) Is there a place inside your home where you can wash your hands?*

( ) Yes

( ) No

#### 32) Is there a place outside but next to your home where you can wash your hands?*

( ) Yes

( ) No

#### 33) Is there a place near the latrine where you can wash your hands?*

( ) Yes

( ) No

#### 34) Does someone have to carry (eg. buckets) water used for washing hands to the house from another location?*

( ) Yes

( ) No

#### 35) Since the beginning of last year until now, has your supply of water for washing hands changed in any of the following ways?*

|  | **Yes** | **No** |
| --- | --- | --- |
| Volume of water normally available has increased | ( ) | ( ) |
| Quality of water normally available has improved | ( ) | ( ) |
| Reliability of water supply has improved | ( ) | ( ) |
| Number of water sources can access has increased | ( ) | ( ) |

#### 36) Does your workplace have places to wash your hands?*

( ) Yes

( ) No

## Handwashing practices

**Logic: Show/hide trigger exists.**

#### 37) How many times per day do you wash your hands?*

( ) not at all

( ) 1-2 times

( ) 3-5 times

( ) 6-10 times

( ) 11-20 times

( ) more than 20 times

**Logic: Hidden unless: #37 Question "How many times per day do you wash your hands?" is one of the following answers ("1-2 times","3-5 times","6-10 times","11-20 times","more than 20 times")**

#### 38) How long do you spend washing your hands each time?*

( ) less than 10 seconds

( ) 10 to 30 seconds

( ) more than 30 seconds

#### 39) Do you usually wash your hands in these situations?*

|  | **Yes** | **No** |
| --- | --- | --- |
| Before eating meals | ( ) | ( ) |
| After eating meals | ( ) | ( ) |
| After going to the toilet | ( ) | ( ) |
| Before touching sick people | ( ) | ( ) |
| After touching sick people | ( ) | ( ) |
| After coming back home | ( ) | ( ) |
| Before going to bed | ( ) | ( ) |
| Whenever they look dirty | ( ) | ( ) |

## Wearing & washing face masks

#### 40) Since the beginning of last year until now, did you usually put on a face mask in these situations?*

|  | **Yes** | **No** |
| --- | --- | --- |
| Before entering crowded places | ( ) | ( ) |
| Before meeting and talking with strangers | ( ) | ( ) |
| Before approaching very old people | ( ) | ( ) |
| Before going near sick people | ( ) | ( ) |
| Whenever leave home | ( ) | ( ) |
| Whenever I had a cough | ( ) | ( ) |
| When air quality was poor | ( ) | ( ) |

#### 41) Which types of masks have you worn? *

[ ] Reusable cloth mask

[ ] Disposable surgical mask

[ ] PM2.5 mask

[ ] Other - Write In: _________________________________________________

#### 42) Since the beginning of last year until now, did you ever not have enough clean water to wash your face mask when you wanted to do so?*

( ) Yes

( ) No

#### 43) Since the beginning of last year until now, did you ever wash your face mask with water that you thought was dirty or unsafe?*

( ) Yes

( ) No

#### 44) Since the beginning of last year until now, did you ever stop washing your face masks with water from a source because it tasted, looked or smelt bad?*

( ) Yes

( ) No

## Awareness of COVID-19

Page exit logic: Skip / Disqualify LogicIF: #45 Question "Have you ever heard of COVID-19 (or other local terms)?" is one of the following answers ("No") THEN: Jump to [page 21 - Contact information](#Page9)

**Logic: Show/hide trigger exists.**

#### 45) Have you ever heard of COVID-19 (or other local terms)?*

( ) Yes

( ) No

## Understanding of COVID-19

**Logic: Hidden unless: #45 Question "Have you ever heard of COVID-19 (or other local terms)?" is one of the following answers ("Yes")**

#### 46) Are the following statements about COVID-19 true (correct)?*

|  | **Yes** | **No** |
| --- | --- | --- |
| You can be infected by touching a surface previously touched by someone who has COVID-19 | ( ) | ( ) |
| Common symptoms of COVID-19 include fever and a dry cough | ( ) | ( ) |
| COVID-19 is spread by mosquito bites | ( ) | ( ) |
| Wearing a facemask reduces risk of getting COVID-19 | ( ) | ( ) |
| Washing your hands frequently reduces risks of getting COVID-19 | ( ) | ( ) |
| Once infected, young adults are more susceptible than the elderly | ( ) | ( ) |

#### 47) What, for you, were important sources of information about COVID-19? (You may select more than one box)*

[ ] Radio

[ ] Television

[ ] Newspapers

[ ] Government posters

[ ] Social media

[ ] Community leaders

[ ] Health workers

[ ] Neighbors

[ ] Relatives

[ ] Household members

[ ] Internet

[ ] Government official

## COVID-19 outbreak and mobility

#### 48) Since the beginning of last year until now, did the government lockdowns, curfews, or other restrictions on movement in response to the COVID-19 outbreak, have a significant impact on your movements in any of the following ways?*

|  | **Yes** | **No** |
| --- | --- | --- |
| Made it more difficult to travel to buy food and other necessities | ( ) | ( ) |
| Made it more difficult to visit family, relatives or friends | ( ) | ( ) |
| Made it harder or impossible to travel to work | ( ) | ( ) |
| Made it harder to go and get food from fields, forest or waterbodies | ( ) | ( ) |
| Made it more difficult to go to the hospital or clinic for treatment | ( ) | ( ) |

#### 49) In which months did the COVID-19 outbreak significantly impact your mobility? (You may select more than one box)*

[ ] Not applicable (no impact)

[ ] January 2020

[ ] February 2020

[ ] March 2020

[ ] April 2020

[ ] May 2020

[ ] June 2020

[ ] July 2020

[ ] August 2020

[ ] September 2020

[ ] October 2020

[ ] November 2020

[ ] December 2020

[ ] January 2021

## COVID-19 outbreak & work

#### 50) Since the beginning of last year until now, did the COVID-19 outbreak have significant impacts on any of these aspects of your livelihood or work?*

|  | **Yes** | **No** |
| --- | --- | --- |
| Reduced my income | ( ) | ( ) |
| Made it harder to sell things | ( ) | ( ) |
| Increased cost of inputs | ( ) | ( ) |
| Reduced demand for my services | ( ) | ( ) |
| Did fewer days of paid work | ( ) | ( ) |

#### 51) In which months did the COVID-19 outbreak significantly disrupt your livelihood? (You may select more than one box)*

[ ] Not applicable (no impact)

[ ] January 2020

[ ] February 2020

[ ] March 2020

[ ] April 2020

[ ] May 2020

[ ] June 2020

[ ] July 2020

[ ] August 2020

[ ] September 2020

[ ] October 2020

[ ] November 2020

[ ] December 2020

[ ] January 2021

## COVID-19 outbreak & transport services

#### 52) Since the beginning of last year until now, did the COVID-19 outbreak have significant impacts on your ability to access these services?*

|  | **Yes** | **No** |
| --- | --- | --- |
| Goods I wanted were not available in the market | ( ) | ( ) |
| Officials were slower in responding to requests for assistance | ( ) | ( ) |
| Public transport services were reduced | ( ) | ( ) |
| Buyers of agricultural products did not come to pick up harvests | ( ) | ( ) |
| Ordered goods were not collected | ( ) | ( ) |
| Home deliveries of food and drinks became convenient but more expensive | ( ) | ( ) |

#### 53) In which months did the COVID-19 outbreak significantly disrupt your access to goods and services? (You may select more than one box)*

[ ] Not applicable (no impact)

[ ] January 2020

[ ] February 2020

[ ] March 2020

[ ] April 2020

[ ] May 2020

[ ] June 2020

[ ] July 2020

[ ] August 2020

[ ] September 2020

[ ] October 2020

[ ] November 2020

[ ] December 2020

[ ] January 2021

## COVID and handwashing

#### 54) At any time, did the COVID-19 outbreak have any of the following impacts on your handwashing behavior (compared to before outbreak)?*

|  | **Yes** | **No** |
| --- | --- | --- |
| Wash hands more thoroughly | ( ) | ( ) |
| Wash hands more frequently | ( ) | ( ) |
| Use soap more frequently | ( ) | ( ) |
| Use alcoholic gel more frequently | ( ) | ( ) |
| Wash hands in more situations | ( ) | ( ) |

#### 55) Did the COVID-19 outbreak have any of the following impacts on your household's water supplies for handwashing?*

|  | **Yes** | **No** |
| --- | --- | --- |
| Switched to cleaner sources | ( ) | ( ) |
| Increased number of sources | ( ) | ( ) |
| Improved delivery system | ( ) | ( ) |
| Increased storage | ( ) | ( ) |
| Made facilities more convenient | ( ) | ( ) |

## COVID and drinking water

#### 56) At any time, did the COVID-19 outbreak have any of the following impacts on your drinking water practices (compared to before outbreak)?*

|  | **Yes** | **No** |
| --- | --- | --- |
| Drank less bottled water | ( ) | ( ) |
| Had less money could spend on drinking water | ( ) | ( ) |
| Drank water from less safer sources more often | ( ) | ( ) |
| Boiled water prior to drinking more often | ( ) | ( ) |

#### 57) Did the COVID-19 outbreak disrupt delivery of drinking water to your household or community?*

( ) Yes

( ) No

#### 58) Did the COVID-19 outbreak make it more expensive to buy drinking water?*

( ) Yes

( ) No

#### 59) Were the impacts of the COVID-19 outbreak at any time made worse by any of these other conditions affecting drinking water supplies?*

|  | **Yes** | **No** |
| --- | --- | --- |
| Drought conditions | ( ) | ( ) |
| Flood events | ( ) | ( ) |
| Conflicts over water | ( ) | ( ) |
| Saline intrusion | ( ) | ( ) |
| Water pollution episodes | ( ) | ( ) |

## COVID and masks

#### 60) Did the COVID-19 outbreak have any of the following impacts on you wearing and washing masks?*

|  | **Yes** | **No** |
| --- | --- | --- |
| Wore a mask more often outside the home | ( ) | ( ) |
| Wore a mask more often at home | ( ) | ( ) |
| Changed masks more frequently | ( ) | ( ) |
| Washed masks more thoroughly | ( ) | ( ) |
| Washed masks more frequently | ( ) | ( ) |

## COVID and social relations

#### 61) Did you ever receive help in dealing with the impacts of COVID-19 from any of the following people? (You may select more than one box)*

[ ] Local government officials

[ ] Other household members

[ ] Neighbors (other households in your community)

[ ] Family or relatives in your community

[ ] Family or relatives living elsewhere

[ ] Other - Write In: _________________________________________________

#### 62) Did you ever help other households in your community cope with the COVID-19 impacts?*

( ) Yes

( ) No

#### 63) Do these statements about the impacts of the COVID-19 outbreak apply to  you?*

|  | **Yes** | **No** |
| --- | --- | --- |
| Brought our family closer together | ( ) | ( ) |
| Improved cooperation with others in the community | ( ) | ( ) |
| Made me more scared of strangers | ( ) | ( ) |
| Increased tension or conflict within the household | ( ) | ( ) |

## Gender relations

#### 64) Were women or men in your household impacted more by the following conditions arising from the COVID-19 outbreak?*

|  | **Women** | **Men** | **Same** | **Neither** |
| --- | --- | --- | --- | --- |
| Restrictions on mobility | ( ) | ( ) | ( ) | ( ) |
| Closure of businesses | ( ) | ( ) | ( ) | ( ) |
| Reduction in personal income | ( ) | ( ) | ( ) | ( ) |
| Reduction in household income | ( ) | ( ) | ( ) | ( ) |
| Stressful relations in the household | ( ) | ( ) | ( ) | ( ) |
| Stressful relations in the community | ( ) | ( ) | ( ) | ( ) |
| Closure of schools | ( ) | ( ) | ( ) | ( ) |

#### 65) Were women or men in your household more likely to do the following tasks?*

|  | **Women** | **Men** | **Same** | **Neither** |
| --- | --- | --- | --- | --- |
| Fetch water for use in the home | ( ) | ( ) | ( ) | ( ) |
| Wash dishes | ( ) | ( ) | ( ) | ( ) |
| Prepare and cook meals | ( ) | ( ) | ( ) | ( ) |
| Clean inside the house | ( ) | ( ) | ( ) | ( ) |
| Buy drinking water | ( ) | ( ) | ( ) | ( ) |
| Maintain facilities for hand-washing | ( ) | ( ) | ( ) | ( ) |
| Take care of eldery members | ( ) | ( ) | ( ) | ( ) |
| Take care of sick or injured members | ( ) | ( ) | ( ) | ( ) |
| Make decisions related to water supplies | ( ) | ( ) | ( ) | ( ) |

## How you coped? What should be done?

#### 66) To cope with the impacts of the COVID-19 outbreak, did you ever do any of the following?*

|  | **Yes** | **No** |
| --- | --- | --- |
| Borrow money from others | ( ) | ( ) |
| Receive money as compensation | ( ) | ( ) |
| Accept food gifts, aid or donation | ( ) | ( ) |
| Seek alternative sources of income | ( ) | ( ) |
| Request improved access to water | ( ) | ( ) |

#### 67) Would the following actions help your or other households in your community better cope with the impacts of the COVID-19 outbreak?*

|  | **Yes** | **No** |
| --- | --- | --- |
| Reduce interest rates on loans | ( ) | ( ) |
| Provide direct financial aid for low income households | ( ) | ( ) |
| Provide financial aid for the unemployed | ( ) | ( ) |
| Provide new jobs for those who became unemployed | ( ) | ( ) |
| Improve the reliability of water supplies to households | ( ) | ( ) |
| Improve the quality of water supplied so it is safe to drink | ( ) | ( ) |
| Improve dry season water storage capacity of community | ( ) | ( ) |
| Provide low-income households with free alchol gel for washing hands | ( ) | ( ) |
| Provide low-income households with free face masks | ( ) | ( ) |

## Contact information

### 68) What is your contact information?

Name of interviewee*: _________________________________________________

Phone number: _________________________________________________

#### Other - please specify

____________________________________________

____________________________________________

____________________________________________

____________________________________________

Name of interviewer*: _________________________________________________

## Thank You!
